# Red Light Optogenetics in Neuroscience

**DOI:** 10.3389/fncel.2021.778900

**Published:** 2022-01-03

**Authors:** Kimmo Lehtinen, Miriam S. Nokia, Heikki Takala

**Affiliations:** ^1^Department of Biological and Environmental Science, Nanoscience Center, University of Jyväskylä, Jyväskylä, Finland; ^2^Department of Psychology, University of Jyväskylä, Jyväskylä, Finland; ^3^Centre for Interdisciplinary Brain Research, University of Jyväskylä, Jyväskylä, Finland

**Keywords:** optogenetics, neuroscience, brain, neuron, near-infrared, opsin, phytochrome

## Abstract

Optogenetics, a field concentrating on controlling cellular functions by means of light-activated proteins, has shown tremendous potential in neuroscience. It possesses superior spatiotemporal resolution compared to the surgical, electrical, and pharmacological methods traditionally used in studying brain function. A multitude of optogenetic tools for neuroscience have been created that, for example, enable the control of action potential generation via light-activated ion channels. Other optogenetic proteins have been used in the brain, for example, to control long-term potentiation or to ablate specific subtypes of neurons. In *in vivo* applications, however, the majority of optogenetic tools are operated with blue, green, or yellow light, which all have limited penetration in biological tissues compared to red light and especially infrared light. This difference is significant, especially considering the size of the rodent brain, a major research model in neuroscience. Our review will focus on the utilization of red light-operated optogenetic tools in neuroscience. We first outline the advantages of red light for *in vivo* studies. Then we provide a brief overview of the red light-activated optogenetic proteins and systems with a focus on new developments in the field. Finally, we will highlight different tools and applications, which further facilitate the use of red light optogenetics in neuroscience.

## Introduction

Optogenetics is a field of science focusing on controlling cellular functions with light-sensing proteins (Deisseroth et al., 2006). Neuroscience has always been the major target for optogenetic systems, and they are well established as neuroscientific research methods (Mahmoudi et al., 2017). The goal in most neuroscientific studies conducted in living animals *in vivo* is to find out the role of a certain structure or cell type in cognition and behavior. To get to this goal, researchers try to manipulate the function of a specific cell type within a specific brain structure during a specific time, while maintaining the rest of the brain as intact as possible. For this purpose, the spatiotemporal precision of optogenetics is superior compared to traditional research methods in neuroscience. The temporal resolution of optogenetics is dependent on light exposure (i.e., turning the light on or off). The spatial resolution is a combination of gene technology and the accurate focusing of the light on the target. Gene technology ensures the expression of the optogenetic system in the specific cells and the localization in targeted cellular compartments (Ziegler and Möglich, 2015).

**FIGURE 1:**
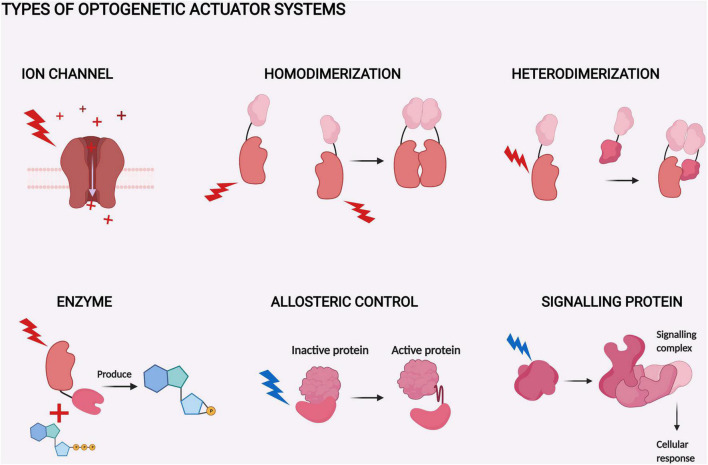
Types of optogenetic actuator systems. The six major actuator systems and the example studies related to them pertain to ion channels (Klapoetke et al., 2014), homodimerization (Schmidl et al., 2019), heterodimerization (Kaberniuk et al., 2016), enzyme activity (Gasser et al., 2014), allosteric control (Strickland et al., 2008), and signaling proteins (Kim et al., 2005). The components of the systems with similar function are depicted with a similar shape. The shapes are not accurate depictions of the actual proteins and are not to scale. The illumination of each actuator is depicted as lightning and colored according to each system. The figure was created with BioRender.com.

Optogenetic systems are based on light-sensitive proteins which can be divided into indicators and actuators (Rost et al., 2017). Indicators are utilized to observe different cellular processes, as their optical properties are sensitive to changing cellular parameters, like pH and Ca^2+^ (Germond et al., 2016). Our review focuses on actuators. Actuators are mainly natural sensory photoreceptors used by various life forms to adjust their behavior to the present light conditions. Most actuators require an extrinsic chromophore to function. Chromophores are small molecules capable of absorbing light at a certain wavelength range between UV and infrared light. In optogenetic systems, absorbance by the chromophore triggers conformational changes in the actuator, which in turn usually activates the function. This function can be based on several mechanisms, like light-induced dimerization, ion channeling, or enzymatic activity (Figure 1). Modification of the actuators through protein engineering enables control of a vast variety of different cellular processes (Rost et al., 2017).

One of the major obstacles in optogenetics is the fact that light does not easily penetrate biological tissues (Chernov et al., 2017). Biological tissues have several different structures and molecules which readily scatter, absorb, or reflect visible light (Ash et al., 2017). *In vivo* studies suffer greatly from this light penetration problem. The problem can be circumvented, for example, by intracranial light sources that significantly increase the invasiveness of the procedure, and by using higher light intensities that damage the brain tissue (Hu et al., 2017; Pisanello et al., 2018). Different wavelengths of light have different abilities to penetrate tissues. Generally, the light penetration increases as the energy of the light decreases. For example, in the visible light spectrum, tissue penetration by blue light is lower compared to red light (Ash et al., 2017). Most of the existing optogenetic systems still utilize blue light (Losi et al., 2018).

The advances of red light in optogenetics can alleviate the light penetration problem (Chernov et al., 2017). In this review, we first describe the advantages of red light-operated optogenetic actuators compared to blue light-based systems in neuroscience. Then we describe the current state of red light optogenetics in detail and highlight the recent advances in the field. Lastly, we introduce attempts to improve the applicability of optogenetics in neuroscientific research.

## Red Versus Blue

In the first section, we compare red light to blue light in terms of their physical and biological properties for neuroscientific optogenetic *in vivo* studies. The reason for the comparison is to highlight the beneficial properties of red light. A comparison is made with blue light-operated systems, which comprise the majority of the published optogenetic systems. Three relevant matters to be compared between optogenetics utilizing blue vs. red light are light penetration properties, possible side effects, and chromophore availability.

**FIGURE 2:**
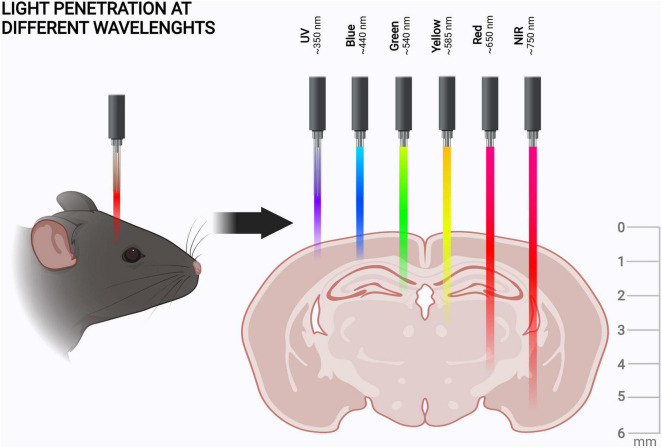
Tissue penetration of different wavelengths of light. Tissue penetration is illustrated from the coronal view of the rodent brain. The light penetration depths, scales, and shown wavelengths are indicative (Jacques, 2013; Diem et al., 2016; Ash et al., 2017). The figure was created with BioRender.com.

It should also be noted that shorter wavelengths of visible light have lower tissue penetration than red light (Figure 2). Here we define the wavelength ranges for blue (∼430–500 nm), red (∼630–710 nm), and near-infrared (NIR) light (∼710–1400 nm), as depicted in Figure 2. In discussing specific illumination schemes, we consider red and NIR light separately to inform about the specific illumination ranges. Once red light in optogenetics is discussed in general, red and NIR wavelengths are not specified separately.

### Light Penetration Properties

In *in vitro* studies, illumination is relatively easy, as the light path is not obstructed. However, attaining sufficient illumination to activate optogenetic systems *in vivo* can be challenging. Various molecules and structures in tissues scatter, absorb, and reflect light differently, which leads to large variations in light penetration (Jacques, 2013). When targeting the brain for illumination of optogenetic systems, the biological tissues to be penetrated are the skin and bone with connective and neural tissues underneath and blood circulating through them.

Scattering is the most significant factor diminishing light penetration in biological tissues. First, scattering of light is wavelength-dependent, with shorter wavelengths scattering more. Therefore, the tissue penetration of blue light is low compared to red light (Al-Juboori et al., 2013; Azimipour et al., 2014; Yona et al., 2016). Second, due to constant variation of the refraction index at liquid-lipid interfaces, the composition of the biological tissue affects scattering (Taddeucci et al., 1996; Xu and Alfano, 2005; Jacques, 2013). For example, bone induces less scattering than skin due to its more homogenous structure (Lister et al., 2012; Soleimanzad et al., 2017). Interestingly, scattering of the skull significantly increases as the animal matures and the skull composition becomes more inorganic (Soleimanzad et al., 2017; Zhao et al., 2017). Furthermore, light scattering varies significantly between different brain structures. The main determining factor is the density of cells in the area, as cell nuclei scatter light much more than axons and dendrites (Al-Juboori et al., 2013; Favre-Bulle et al., 2015). However, also the directional organization and myelination of axons increase scattering, for example, in the spinal cord (De Paoli et al., 2020).

Absorption is the other main physical phenomenon decreasing light penetration in biological tissues. Small tissue-specific molecules play an important role in that: skin contains natural photoprotective pigment, melanin, which significantly decreases the penetration of blue light (Jacques and McAuliffe, 1991). Another specific light-absorbing molecule to be considered is blood hemoglobin, which has higher blue absorption than red (Zijlstra et al., 1991). Besides the tissue-specific molecules, fat and water also affect light absorption (Jacques, 2013). Absorption by fat is relatively similar under both red and blue light. Still, absorption characteristics can vary significantly within these wavelength ranges (Hale and Querry, 1973; Anderson et al., 2006). Water mainly absorbs light at longer NIR wavelengths, but the wavelengths applied in optogenetics are in the low absorption ranges (Hale and Querry, 1973; Sordillo et al., 2017).

The third physical factor, reflection, occurs mainly in the skin. However, its total effect on light penetration is minuscule (Lister et al., 2012; Favre-Bulle et al., 2015) and is not therefore considered further here.

Taken together, red and NIR light penetrate both skin and bone better than blue light. For example, red light penetration in skin is 4–5 mm (Ash et al., 2017), whereas for blue light this is only ∼1 mm (Jacques, 2013). Of course, the optical properties of the scalp and the skull can be altered by thinning, or the tissues can be removed altogether and replaced with transparent material to create a window into the brain (Cramer et al., 2021). However, the use of red or NIR light could enable *in vivo* manipulation of the brain in animal models without the need for this, as the mouse scalp is 0.3–0.6 mm (Azzi et al., 2005) and the skull is 0.3–0.4 mm thick (Jacques, 2013; Park et al., 2015). Light penetration into brain tissue itself is dependent on the targeted structure but is generally more effective than in skin (Jacques, 2013). As the maximal sagittal length of the mouse brain is approximately 5–6 mm (Diem et al., 2016), red light can enable non-invasive optogenetic manipulation of even the deepest brain structures in mice (Figure 2). This puts the advantages of red light penetration into perspective, if at first glance they seemed minuscule.

### Possible Side-Effects

The energy transfer from light can cause side effects in living tissues. UV light has long been known to cause phototoxic damage when absorbed, due to the high energy transfer (Hu et al., 2017). This suggests a higher probability of phototoxic effects with blue light in optogenetic experiments, compared to the lower energy of red light. The actual phototoxic effects can be well mitigated by phototoxicity controls and careful illumination protocols, which address the total power applied to the target (Ziegler and Möglich, 2015; Smart et al., 2017). Similarly, absorbance by water in the red and infrared spectral range can heat up the tissue and cause thermal damage (Ash et al., 2017). Again, a well-planned illumination scheme and pulsed light can significantly mitigate the risk of overheating (Ziegler and Möglich, 2015; Zheng et al., 2017).

Natural light reception has emerged as a potential cause of significant disturbance in optogenetic studies. The brain and other non-retinal tissues contain UV and blue light photoreceptors, which apparently can even be activated transcranially (Flyktman et al., 2015; Nissilä et al., 2016; Zhang K. et al., 2020). This could become a problem when interpreting the outcome of experimental manipulation. For example, in optogenetic apoptosis studies (Nihongaki et al., 2014; Hughes et al., 2016; Ji et al., 2019), the activation of cell survival or cell death pathways through natural photoreceptors could protect the target cells from experimental manipulation or cause unwanted damage, respectively (Yoshimoto et al., 2018; de Assis et al., 2020). Blue light can also activate several natural pathways in neural cells even without applied optogenetic actuators (Tyssowski and Gray, 2019).

When it comes to red light, the mechanisms behind photobiomodulation (PBM) can cause similar problems. PBM is a field that applies low-power red light illumination for therapeutic purposes. The mechanisms of PBM are still under debate, but are possibly related to mitochondrial enzymes, the production of reactive oxygen species, and endogenous red light-sensing proteins (Hamblin, 2016). Outside of different survival pathways, transcranial PBM has been shown to modulate neural activity, which was apparent through changes in electroencephalography (EEG) even in humans (Zomorrodi et al., 2019; Wang et al., 2021). To summarize, the effects of both endogenous opsin activity (induced by blue light) and PBM (induced by red light) need to be considered in each study.

### Chromophore Availability

The utility of an optogenetic actuator is highly dependent on the availability of a suitable chromophore (i.e., light-absorbing molecule) in the target. In principle, actuators without a need for an extrinsic chromophore would be ideal, but the existing actuators with intrinsic chromophores utilize only strongly scattering and phototoxic UV light (Wu et al., 2012; Zhou et al., 2012). Most of the blue light-sensing actuators utilize different flavin molecules (Losi et al., 2018). Flavins are essential to major redox reactions, like NADH/NAD metabolism, and are readily available in all cell types (Tomiki and Saitou, 2004). Different widely used opsin proteins are activated by either blue or red light (Nagel et al., 2003; Chuong et al., 2014). Their chromophore, a derivate of vitamin A called retinal, seems to be available in most *in vivo* models in broad utilization in neuroscience (Deisseroth, 2015).

Most red light-driven optogenetic actuators utilize different bilin molecules as their chromophores, the availability of which varies between the target cells (Chernov et al., 2017). Bacterial phytochromes utilize biliverdin, a breakdown product of heme, which should be available in sufficient concentrations for *in vivo* experiments in most animal models (Tenhunen et al., 1969; Bhoo et al., 2001). Plant phytochromes, cyanobacterial phytochromes, and cyanobacteriochromes (CBCRs) depend on bilins, phytochromobilin, or phycocyanobilin, which are absent from animal cells. Therefore, their utilization in animal studies is limited. The utilization of these phytochromes in animal cells therefore requires the external addition of chromophores or the implementation of a genetically engineered chromophore-producing machinery (Buckley et al., 2016; Uda et al., 2017). For example, phycocyanobilin can be made available in mammalian cells by co-expressing the enzymes heme oxygenase 1 (HO1) and phycocyanobilin:ferredoxin oxidoreductase (PcyA) (Uda et al., 2017).

Blue light-driven optogenetic actuators seem to have a slight edge in chromophore availability. Still, several advances have been made to ease the bilin availability for red light-sensing optogenetic actuators. For example, the genetically engineered production of bilins has been further improved (Kyriakakis et al., 2018; Uda et al., 2020), and biliverdin-utilizing CBCRs have been either discovered or created by mutations (Fushimi et al., 2019; Moreno et al., 2020; Tachibana et al., 2021).

## Red Light-Sensing Optogenetic Actuators

**FIGURE 3:**
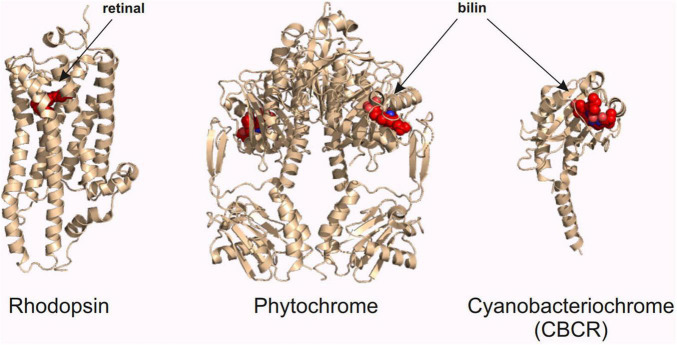
Red light optogenetic actuator classes. Example structures of the three classes of the red light optogenetic actuators. Note that only the photosensory fragment of each protein is shown. The structures are to scale relative to each other. Each structure was derived from the following Protein Data Bank (Berman et al., 2000) coordinates: 3AYN (rhodopsin) (Murakami and Kouyama, 2011), 4O0P (phytochrome) (Takala et al., 2014), and 6UV8 (cyanobacteriochrome) (Bandara et al., 2021). The figure was created with the PyMOL Molecular Graphics System version 2.3.3 (Schrödinger, LLC).

As stated in the previous chapters, red light has its advantages in optogenetic research in neuroscience. In this chapter, we introduce three classes of red light-sensing optogenetic actuators: rhodopsins, phytochromes, and cyanobacteriochromes (Figure 3). We first give a short introduction of the structure and function of these proteins and then present examples of how to create improved optogenetic actuators. We also highlight the development of specific binding partners of these actuators, which constitute an important part of the red light optogenetic toolbox.

### Rhodopsins

The field of optogenetics has been built on neuroscientific research with opsins (Deisseroth, 2015). The first optogenetic systems were based on opsins and new applications are still routinely published (Zemelman et al., 2002; Essig et al., 2021; Zhang et al., 2021). Opsins are heptahelical transmembrane proteins that are called rhodopsins once covalently bound to their chromophore, retinal (Palczewski et al., 2000; Figure 3). Optogenetics has mainly utilized opsin types that act as light-activated ion channels and ion pumps (Deisseroth, 2015). These opsins show specificity to certain ions, based on the size and the charge of the channel (Luecke et al., 1999; Wietek et al., 2014). Different opsin channels match perfectly with the electrical signal transmission of the neurons in the central nervous system. A positive or negative ion flow into a neuron changes the membrane potential and can either facilitate or inhibit the firing of an action potential (Vierock et al., 2021). Besides ion channels, some rhodopsins can act as light-activated G-protein coupled receptors or enzymes, like phosphodiesterases (Dixon et al., 1986; Yoshida et al., 2017). These rhodopsins could be utilized in the manipulation of signaling pathways or cellular concentrations of second messengers with light (Jastrzebska et al., 2016; Tian Y. et al., 2021).

### Phytochromes and Cyanobacteriochromes

Phytochromes are natural dimers of two photosensory modules (Figure 3), which are accompanied by enzymatic effector domains (Karniol et al., 2005). The photosensory module is composed of a PAS (Period/ARNT/Single-minded), a GAF (cGMP phosphodiesterase/adenylyl cyclase/FhlA), and a PHY (Phytochrome-specific) domain, which are linked together by an α-helical backbone (Burgie et al., 2014). The light-activated conformational changes of phytochromes are particularly interesting among optogenetic actuators (Takala et al., 2020). During the conformational changes, PHY domains are moved in relation to each other, accompanied by changes in their secondary structure (Takala et al., 2014, 2018; Björling et al., 2016). The activity of phytochromes, which can be the activity of an attached effector domain or changes in protein interactions, is controlled by these changes in the photosensory module. In optogenetics, these changes are harnessed by different effector domains or binding partners (Dubreuil et al., 2017; Multamäki et al., 2021). The binding partners allow the creation of different localization systems, which, for example, can modulate the local concentrations of the attached signaling proteins (Chernov et al., 2017).

Non-photochromic actuators are proteins that can only be activated with light. Therefore, the duration of their active period is not dependent only on the duration of the light exposure. After the light has been switched off, the non-photochromic actuator will return to the most thermodynamically stable conformation, called a dark state, through dark reversion (i.e., thermal motion) (Ziegler and Möglich, 2015). Different actuators have been designed with desired dark reversion times in relation to biological functions, like submillisecond reversion for neural firing (Klapoetke et al., 2014).

Phytochromes, however, are photochromic proteins, that is, they can be shuttled between active and inactive conformations by different wavelengths of red light (Rockwell et al., 2006). The photochromic actuators therefore allow increased control of the signal length. Most phytochromes adopt a red light-absorbing (Pr) state as their dark state, whereas red light causes them to convert to a far-red light-absorbing (Pfr) state. The Pfr state can then absorb NIR light and return the phytochrome back to the Pr state (Takala et al., 2014). Some phytochromes have adopted the Pfr state as their dark-adapted state, leading to a reverted photocycle in these bathy phytochromes (Rottwinkel et al., 2010). The photochromic capacity of the phytochromes has been only marginally utilized in optogenetics, although it has obvious potential to study different reversible biological functions. For example, photochromic optogenetic apoptosis systems could be used to study anastasis, the natural reversion of the cell death (Tang and Tang, 2018).

Cyanobacteriochromes have been utilized relatively little as optogenetic actuators compared to the other two red light-sensing actuator classes described above. CBCRs are photochromic relatives of the phytochromes, as they are composed of a singular GAF domain and bind a bilin chromophore (Figure 3; Xu et al., 2020). The smaller size and the increased photochromic capabilities can drive their utilization in future research.

### Development of the Proteins

One important way to improve the utilization of red light in optogenetics is to gain more knowledge about the actuator proteins. New knowledge can then be utilized by genetically modifying the protein for more desirable attributes, like lower dark activity. Novel actuators and binding partners can also be found from nature, which can offer unprecedented options for optogenetic control.

New knowledge has been gained on the structure of red light-sensing opsins (Oda et al., 2018) and the activation dynamics of CBCRs (Wang D. et al., 2020; Ruf and Buhrke, 2021). Novel advances with phytochromes include the structural characterization of the plant phytochrome (Tahir ul Qamar et al., 2021) and increased knowledge about signal transduction in the bacterial phytochromes (Böhm et al., 2021). One intriguing discovery has revealed that a bacterial phytochrome is capable of two-photon absorption. Therefore, instead of a single higher-energy photon, two lower-energy photons can be used to drive the conformational changes in bacterial phytochromes (Sokolovski et al., 2021). This means that even NIR light with a long wavelength (∼1200–1300 nm) could be used in phytochrome-based studies.

Different dimerization-based localization systems (Figure 1) are an important subset of the optogenetic toolbox for neuroscience. They can be based on homodimerization (i.e., actuator-actuator interaction) or heterodimerization (i.e., actuator-binding partner interaction). Red light-controlled homodimerization has been mainly based on a cyanobacterial phytochrome Cph1 (Schmidl et al., 2019; Sentürk et al., 2019; Rasoulinejad et al., 2020). Homodimerization systems only need one vector to be transported to the target, which decreases the complexity of the study. Heterodimerization systems usually require two vectors, but the small size of certain binding partners may enable the use of a single vector for both components (Kaberniuk et al., 2021; Kuwasaki et al., 2021).

For red light-controlled heterodimerization systems, the development of binding partners for phytochromes and CBCRs is crucial for attaining improved optogenetic systems. The first systems were based on the plant phytochrome B and its binding partner Phytochrome-Interacting Factor (PIF) (Shimizu-Sato et al., 2002). These systems are still routinely utilized despite the hindrance of their larger size and complicated application due to the non-mammalian chromophore (Raghavan et al., 2020; Fonin et al., 2021). However, successful efforts have been made to decrease the size of PIF and increase its binding affinity to phytochrome (Golonka et al., 2019; Yousefi et al., 2019).

The first bacterial heterodimerization system, which is based on the bacterial phytochrome form *Rhodopseudomonas palustris* (*Rp*BphP1) and its binding to PpsR2, suffers from a large size and self-oligomerization (Kaberniuk et al., 2016). PpsR2 has been improved by determining the crucial structures required for the actuator binding. The result, QPAS1, is three times smaller and does not self-oligomerize (Redchuk et al., 2017). However, the QPAS1 system has been shown to have relatively high dark activity, which significantly hinders its future utilization (Kuwasaki et al., 2021).

Fortunately, two very promising, smaller partners that bind to the photosensory module of a bacterial phytochrome from *Deinococcus radiodurans* have been published: nanoREDs are optimized nanobodies, that is, engineered single-domain antibodies from camelids (Huang et al., 2020); MagRED is an optimized Affibody, that is, a domain of an immunoglobulin-binding bacterial surface protein (Kuwasaki et al., 2021). Direct comparison of nanoREDs and MagRED has demonstrated their superiority to older QPAS1-based system. Both MagRED and nanoREDs are shown to function *in vivo*, although the MagRED-based applications were more complex. The *D. radiodurans* phytochrome has slow dark reversion time, which enables long sustained activation of the optogenetic system (minutes vs. hours). Therefore, their activity is also sustained longer, compared to the QPAS1 system (Huang et al., 2020; Kuwasaki et al., 2021).

The optogenetic toolbox based on CBCRs has remained very light compared to the one based on phytochromes. Jang et al. (2019) have published in *bioRxiv* the first CBCR-based binding systems, collectively termed as BAm. BAm has been optimized from a domain of an albumin-binding bacterial surface protein. Different variants of BAm selectively bind different states of the CBCRs but require green light for the photochromic function. In addition, they have only been shown to function in yeasts (Jang et al., 2019).

Lastly, it is always wonderful to find something completely new: new opsin chloride pumps, RubyACR, have been discovered from aquatic protists. They are now one of the most red-shifted anion-passing opsins, which makes their future application in optogenetics relatively likely (Govorunova et al., 2020). An even more curious finding is the new cationic fungal rhodopsin-guanylyl cyclase, NeoR. It is the only known opsin to be active solely as a hetero-oligomer with two other cyclases of the same species. NeoR is also photochromic, as it is activated by UV light but deactivated by NIR. Interestingly, its NIR-absorbing state is highly fluorescent at NIR wavelengths. The cyclase activity can also be induced by blue light through the oligomerizing opsin (Broser et al., 2020). The application of NeoR for optogenetics can have its problems, but new applications are expected due to its variant qualities.

## Red Light-Sensing Systems and Their Applications

In this section, we focus on red light-sensing actuator systems, which have been published during the last few years. The reason for the selected time period is the excellent review of the older systems by Chernov et al. (2017) and the fact that during this time the utilization of red light in optogenetics has increased rapidly. We have a special focus on the actuator systems already applied in neuroscience, but we also speculate on the potential neuroscientific uses for other systems. Still, it is worth mentioning that new red optogenetic indicators are also constantly developed. For example, a new calcium indicator and a new voltage indicator have been published in the last 2 years (Beck and Yiyang, 2019; Shemetov et al., 2020).

### Opsin-Based Applications

Opsins are the most utilized and developed systems in optogenetics. This also reflects on the volume of new publications with red light-activated opsins (Table 1). Sensory restoration with Chrimson opsins has been one of the most published areas (Bali et al., 2021; Huet et al., 2021). The cation channel Chrimson has variants with faster activation/inactivation kinetics than the original. They are therefore more suited for the high temporal resolution required to coincide with human senses (Klapoetke et al., 2014; Mager et al., 2018). Restoration of hearing and sight can be achieved by light activation of ganglion cells, which normally relay input signal from intact sensory receptors to the brain (Bali et al., 2021; Gauvain et al., 2021).

**TABLE 1:** Red light optogenetics in neuroscientific animal studies.

	Application	Target	Subject	System	References
**Opsin**					
	Sight restoration	Retinal ganglion	Human	ChrimsonR	Sahel et al., 2021
	Sight restoration	Retinal ganglion	Non-human primate	ChrimsonR	Gauvain et al., 2021
	Sight restoration	Retinal ganglion	Mongolian gerbil	ChrimsonR	Huet et al., 2021
	Sight restoration	Retinal ganglion	Mouse	ReaChR	Lindner et al., 2020
	Sight restoration	Retinal ganglion	Mouse	MCO	Batabyal et al., 2020
	Sight restoration	Excitatory neurons in primary visual cortex	Rat	ChrimsonR	Masuda and Takahashi, 2021
	Sight restoration	Inhibitory neurons in primary visual cortex	Rat	eNpHR 3.0	Masuda and Takahashi, 2021
	Hearing restoration	Spiral ganglion	Mouse	f-Chrimson and vf-Chrimson	Bali et al., 2021
	Pain research	Microglia in spinal cord	Mouse	ReaChR	Narcisse et al., 2021
	Pain research	Inhibitory neurons in the anterior cingulate cortex	Mouse	MCO II	Yi et al., 2021
	Epilepsy research	Hippocampal CA3 region	Mouse	Jaws	Farzaneh et al., 2020
	Epilepsy research	Hippocampal inhibitory GABAergic interneurons	Mouse	ChRmine	Chen R. et al., 2020
	Feeding -behavior alteration	Dopamine neurons of the ventral tegmental area	Mouse	ChRmine	Chen R. et al., 2020
	Bidirectional neural activity control	Dopaminergic neurons of the ventral tegmental area	Mouse	ChrimsonR and stGtACR2 (blue)	Li et al., 2021
	Bidirectional neural activity control	GABAergic neurons of the secondary visual cortex	Ferret	BiPOLES	Vierock et al., 2021
	Pupil size control	Locus coeruleus	Mouse	BiPOLES	Vierock et al., 2021
**Phytochrome**					
	Sleep spindle modulation	Ventral posteromedial nucleus and nucleus reticularis	Mouse	Adenylate cyclase ilaM5	Fomicheva et al., 2019

*The table contains in vivo applications for red light optogenetic systems in neuroscience, which are studied in primates and rodents. Only applications developed during 2019–2021 are shown.*

Restoration of sight has also been studied with other intermediate cells and opsins: for example, with the inhibition of certain inhibitory neurons by the red light-sensing chloride pump opsin eNpHR 3.0 (Masuda and Takahashi, 2021). Restoration of hearing is still in its preliminary stages, while restoration of sight has shown utility in non-human primates and even proceeded to clinical trials (Bali et al., 2021; Gauvain et al., 2021; Sahel et al., 2021). The most remarkable advance is the partial restoration of sight in a blind patient with retinitis pigmentosa. This was achieved by ChrimsonR activation of the retinal ganglion cells with light-stimulating goggles. The goggles created a visual stimulus by means of a neuromorphic camera. It was also the first reported case of partial functional recovery in a neurodegenerative disease after optogenetic therapy (Sahel et al., 2021).

Other red light opsins have still been actively present in neuroscientific research. ReaChR and MCO have been utilized in the restoration of sight in mice (Batabyal et al., 2020; Lindner et al., 2020). They have also had interesting utilities in pain research (Narcisse et al., 2021; Yi et al., 2021). Microglia, which are neural macrophages, function partly by ionotropic signaling. Prolonged signaling can induce a further inflammatory response, which has been linked to chronic pain. ReaChR stimulation in the spinal cords of mice has also induced an inflammatory response with chronic neural pain (Narcisse et al., 2021). MCO II has been used for pain modulation based on interfering with pain signaling at the limbic cortex. Activation of the inhibitory neurons in the anterior cingulate cortex has induced reduction in the pain response in mice (Yi et al., 2021). At the same time, the anion opsin Jaws and cationic ChRmine have been used to study epilepsy (Chen R. et al., 2020; Farzaneh et al., 2020). In a study featuring Jaws, mesial temporal lobe epilepsy -mimicking seizures has been induced in rats. Jaws activation in the neurons of the hippocampal CA3 region has nullified the seizures (Farzaneh et al., 2020). ChRmine has been used to activate hippocampal inhibitory GABAergic interneurons in mice, which has significantly shortened the seizures. The most impressive part of the study is that light has been administrated non-invasively with transcranial illumination. In the same study, dopamine neurons of the limbic ventral tegmental area have been activated by ChRmine. This has induced feeding-related behavioral changes in the mice. Here, the transcranial activation of ChRmine has been successfully achieved even at the depth of 7 mm (Chen R. et al., 2020).

### Phytochrome-Based Applications

The majority of the new red light-sensing actuator systems outside of opsins are based on the plant phytochrome B and its binding partner PIF. For example, the T cell function has been studied on the basis of this system (Yousefi et al., 2019, 2021). Recent plant phytochrome-based systems have not been utilized in a neuroscientific context, although some of them still have interesting properties for neuroscience research. Integrins have an important role in regulating neuronal connectivity, like neurite growth (Lilja and Ivaska, 2018). The OptoMatrix–OptoIntegrin system can offer a reversible cell-matrix interaction. It utilizes a small PIF variant, PIFs, attached to αVβ3-integrin. After red light illumination, cells expressing the fusion protein on their surface are bound to the phytochrome-coated glass slices (Baaske et al., 2019). In the future, the system could be utilized, for example, to research the plasticity of the brain through controlling axon growth. Bacteriobots can be utilized to release cargo, like nanoparticles or drugs, in precise locations. In a study by Sentürk et al. (2020), *Escherichia coli* was coated with phytochrome and polystyrene particles with PIF6. Red and NIR light illumination was successfully used to attach and release the cargo as desired *in vitro* (Sentürk et al., 2020). In neuroscientific research, this new method could help achieve more spatiotemporally precise drug administration.

**FIGURE 4:**
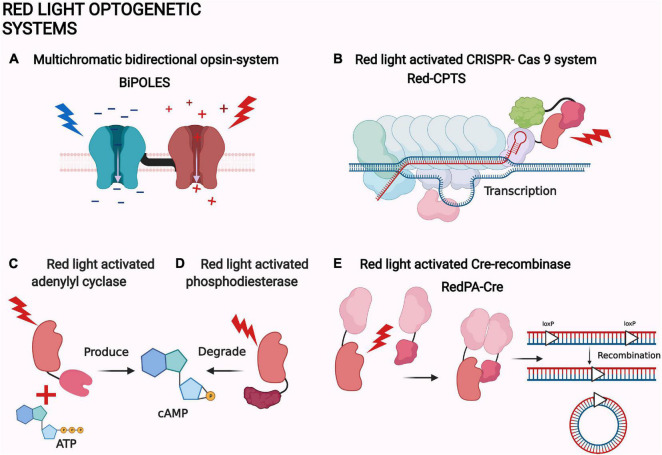
Examples of red light optogenetic systems. **(A)** Multichromatic directional opsin system BiPOLES (Vierock et al., 2021). **(B)** Red light-activated CRISPR-Cas 9 system Red-CPTS (Kuwasaki et al., 2021). **(C)** Red light-activated adenylyl cyclase (Fomicheva et al., 2019). **(D)** Red light-activated phosphodiesterase (Stabel et al., 2019). **(E)** Red light-activated Cre-recombinase RedPA-Cre (Kuwasaki et al., 2021). The same protein components are depicted with a similar shape in each panel. The shapes are not accurate depictions of the proteins and are not to scale. The illumination of each actuator is depicted as lightning and colored according to each system. The figure was created with BioRender.com.

Instead of plant phytochromes, recent bacterial phytochrome-based systems have been utilized in neuroscience. In the most relevant study, new phytochrome-activated adenylate cyclases were designed (Figure 4C and Table 1; Fomicheva et al., 2019). Adenylate cyclases produce the second messenger cAMP, which is an important signaling molecule for a variety of biological functions (Khannpnavar et al., 2020). In the study by Fomicheva et al. (2019), the best performing cyclase, ilaM5, was transfected into the thalamic ventral posteromedial nucleus and the nucleus reticularis in mice. The cyclase was then activated using transcranial NIR light illumination. The activation of the system reversibly suppresses the formation of cAMP-associated electrophysiological events in the brain called sleep spindles. These oscillations have been associated with memory formation. Unfortunately, this application has had problems with dark activity and the suppression of the other signals. Still, the system has potential to be utilized in memory research after optimization (Fomicheva et al., 2019).

The photosensory module from the *Deinococcus radiodurans* phytochrome has also been utilized by other groups to create different cyclases (Etzl et al., 2018; Fomicheva et al., 2019; Kaberniuk et al., 2021). Etzl et al. (2018) created novel optogenetic guanylate and adenylate cyclases from which the latter were utilized to alter the locomotion of *Caenorhabditis elegans* through their cholinergic motor neurons (Etzl et al., 2018). In the iLIGHT system by Kaberniuk et al. (2021), the photosensory module was optimized and linked to diguanylyl cyclase. It was utilized in primary neurons and *in vivo* for transcription modulation in mice (Kaberniuk et al., 2021).

Phosphodiesterases are the natural counterparts of the adenylate cyclases in controlling the second messenger concentrations (Figure 4D; Francis et al., 2011). Therefore, red light-controlled cAMP production and destruction has an intriguing potential for neuroscience. By coincidence, new and improved actuators with phosphodiesterase activity have also been created (Stabel et al., 2019).

CRISPR-Cas9 is the most precise and versatile transcription system in cell biology. The systems have been modified from the bacterial defense mechanism and are capable of single gene transcription, deletion, and even epigenetic modifications (Adli, 2018). Consequently, the first NIR light-activated CRISPR-Cas9 has recently been created (Shao et al., 2018). The NIR light-activated system FACE is based on the phytochrome-controlled diguanylyl cyclase activity, which initiates the production of the transcription factor PFRLx. The induced production of the transactivator complex leads to the formation of the dCas9 transcription complex. The system has been utilized in mice, but more interestingly, it has been used to turn iPSCs stem cells into neurons by a targeted expression of the neuronal transcription factor (Shao et al., 2018).

Intriguingly, the bacterial phytochrome-based MagRED system has also been utilized to create a red light-activated CRISPR-Cas9 (Figure 4B). The system is also based on the transactivator complex formation, but with a direct localization of the MagRED attached complex protein p65-HSF1. The system is called Red-CPTS and has only been used *in vitro*. The other interesting application of MagRED is the red light-activated Cre-recombinase named RedPA-Cre (Figure 4E; Kuwasaki et al., 2021). Cre-recombinases are bacteriophage-derived recombinases which can identify specific DNA sequences called loxP. After locating the loxP sites, recombinase can excise, turn, or move the immediate sequences between or next to them (Duyne, 2015). RedPA-Cre is based on a split-protein approach, where the N- and C-terminal ends of the split-Cre are localized back together by light (Figure 4E). This system has been used to regulate the activity of the predetermined gene *in vitro* (Kuwasaki et al., 2021).

The red light-activated Cre-recombinases have been published relatively often. The plant phytochrome-based split-Cre system, CreLite, has been utilized in zebrafish embryos (Yen et al., 2020). A NIR-light activated recombinase system, FISC, has also been published (Wu et al., 2020). The system is dependent on the natural split proteins attached to the split-Cre. Natural split proteins are encoded by separate genes, which after translation spontaneously dimerize to create a functional protein (Iwai et al., 2006). The light regulation of the system has been achieved by the same transcription regulation as for the NIR light-activated CRISPR-Cas9 system FACE. The system has been successfully utilized *in vivo* in mouse liver (Wu et al., 2020).

### Multichromatic Approaches

Red light-regulated optogenetic systems can be utilized alongside other optogenetic systems operated through the use of different wavelength light, as long as their absorption spectra do not overlap. Therefore, various multichromatic two-actuator approaches have been actively developed. For example, several bidirectional opsin systems have been published that allow both activation and inhibition of the same neuron. The new systems are based on red light activation of the opsin Chrimson and blue light inhibition of an anion opsin. Expression of both actuators in the same neuron facilitates the precise control of action potential generation, as reported by two studies in *BioRxiv* (Li et al., 2021; Mermet-Joret et al., 2021). This approach has been used to control, for example, dopaminergic neuron activity in the ventral tegmental area in behaving mice (Li et al., 2021).

A bidirectional system with two linked opsins, BiPOLES, has also been created (Figure 4A; Vierock et al., 2021). The requirement for only one vector creates an advantage for the BiPOLES. It has been used to control pupil size in mice through the locus coeruleus and GABAergic neuron activity in the secondary visual cortex of ferrets (Vierock et al., 2021). Another implementation of multichromatic systems in one cell group relates to the regulation of neurotrophin function *in vitro*: The photosensory module of *D. radiodurans* phytochrome has been used to activate neurotrophin tyrosine kinases in neurons. This NIR light-activated signaling has been further controlled by attaching the system to the membrane-bound blue light-activated localization system LOV2pep. The idea behind this additional blue light control is the amplified signaling at the membrane compared to the cytosol (Leopold et al., 2019). Multichromatic approaches are also well suited for the simultaneous use of an actuator and an indicator. For example, phytochrome-activated tyrosine kinase signaling has been imaged with a green fluorescent calcium indicator (Leopold et al., 2020).

Several other multichromatic approaches may have future uses in neuroscience. For example, proliferation and survival pathways regulated by AKT/ERK signaling are of crucial importance in neural development and function (Wang L. et al., 2017). In an interesting two-system approach, OptoAKT can be kept active by constant blue light illumination. During the attained constant AKT signaling, OptoSOS controls the ERK signaling by the photochromic control of the plant phytochrome B (Kramer et al., 2021). Both of the above systems are based on localizing the actuator-attached signaling protein on the plasma membrane by a membrane-associated binding partner (Johnson et al., 2017; Wang Q. et al., 2020).

As another example, the idea of a light-responsive cell sorting system could be used to manipulate neuronal development. In an *in vitro* application by Rasoulinejad et al. (2020), cells expressed either homodimerizing blue light-activated VVD or homodimerizing red light-activated phytochrome Cph1. Light-activated dimerization of both systems gathered the cells into two separate multicellular tissue-like structures (Rasoulinejad et al., 2020).

Stray light is a thing to be cautious of in optogenetic applications. The effect of stray light can be diminished, for example, by adjusting the general illumination of the laboratory. Photochromatic systems, like phytochromes, also enable the reversion of possible stray light activation (Kramer et al., 2021). Multichromatic systems can also alleviate the effects of the unwanted illumination. As an example, a multichromatic transcription system developed for plant optogenetics could be used as a template for future applications in neuroscience. The system is based on a repressor protein associated with a blue light actuator, which allows for constant repression under white light. Transcription is activated with red light-induced phytochrome-PIF6 interaction, which brings together the components of a split transcription factor (Ochoa-Fernandez et al., 2020).

## Methods Aiding Optogenetics in Neuroscience

In this section, we are taking a closer look at different methods that have improved or could improve the future optogenetic research with red light. Technical advancements in closely related fields, like robotics, imaging, and biochemistry, have made optogenetics a more and more applicable research approach in the field of neuroscience (Kim et al., 2017). The cornerstones of *in vivo* optogenetics are the transfection of the optogenetic system and the illumination of the target. Improvements in these areas lead to (1) more specific expression of the optogenetic actuators in the desired brain cells, and (2) more accurate manipulation of the system in the brain.

### Transfection

A precise injection of a viral vector to a certain brain area is the main way to deliver the optogenetic system to the target cells. Viral vectors are replication-deficient viral particles, which can carry the DNA of the system into the target cells by infecting them. Injection protocols are constantly evolving to ensure accurate and safe delivery. For example, imaging dyes and magnetic resonance imaging (MRI) are used to ensure the accuracy of the injection (Fredericks et al., 2020). At the other end, a brain-wide expression can be achieved by systemic delivery (i.e., through circulation) (Huang et al., 2021). Delivery into the whole brain can be enhanced by ultrasound, which can be used to temporarily break down the integrity of the blood-brain barrier and thus allow the virus to enter the brain. Opening of the blood-brain barrier is achieved by lipid-based particles, which can be made to oscillate in the capillaries of the target area (Wang S. et al., 2017). Another way to achieve tissue-wide expression is the convection method, where the viral lysate is driven into the cells by a high-pressure injection (Yu et al., 2017). This method has been used to deliver opsins into the cortex of rhesus macaques (Khateeb et al., 2019). Interestingly, viral vectors have also been successfully utilized for tissue-wide expression. These adeno-associated virus (AAV) variants are effectively able to cross the blood-brain barrier, which allows their application intravenously. Therefore, they have lower invasiveness compared to other methods (Deverman et al., 2016; Chan et al., 2017).

Research into viral vectors can give an important indication of their use with a specific target. Several comparative studies of different vectors have been conducted to determine which is best suited for specific *in vivo* targets (Wu et al., 2017; Kondratov et al., 2021). More specifically for optogenetics, AAV serotype 1 has been shown to perform best with opsins in pigeons (Rook et al., 2021). New vectors, like AAV9-Retro, a new retrograde (i.e., infecting from axon to soma) variant, have also been developed. This vector has been shown to pass the blood-brain barrier naturally in mice (Lin et al., 2020). Also, non-pathogenic primate viruses, foamy viruses, have interestingly been used as vectors in the hippocampal structures of mice (Counsell et al., 2018).

A red light-controllable optogenetic viral vector, OptoAAV, has also been developed. The system is composed of an AAV2 vector attached to PIF6 and an engineered antibody-mimicking DARPin attached to plant phytochrome B. The selectivity of the system is based on the specific cell surface protein binding of the used DARPin and the inactivation of the natural AAV viral tropism, that is, the ability of the virus to infect certain cell types better compared to others (Hörner et al., 2021).

Non-viral delivery can have several advantages over viral vectors. Nanoparticles have a larger gene loading capacity; they are easier to modify and contain a lower risk for an immune reaction (Wang and Huang, 2019). They can also be engineered to respond to different cellular parameters, like pH (Yu C. et al., 2021). As new nanoparticle vectors are constantly being created, other non-viral methods have also been reported (Zohri et al., 2021). Non-contact methods are interesting novel applications for transfection (Schubert et al., 2017). One non-contact method has been used to deliver the red-light opsin MCO into ganglion cells of living mice. The system is based on the surface plasmon resonance of nanorods attached to the cell membrane. After weak NIR light illumination, the heat created by the nanorod resonance disturbs the cell membrane, which allows the freely flowing DNA plasmid to intrude the cell (Batabyal et al., 2020).

### Illumination

**FIGURE 5:**
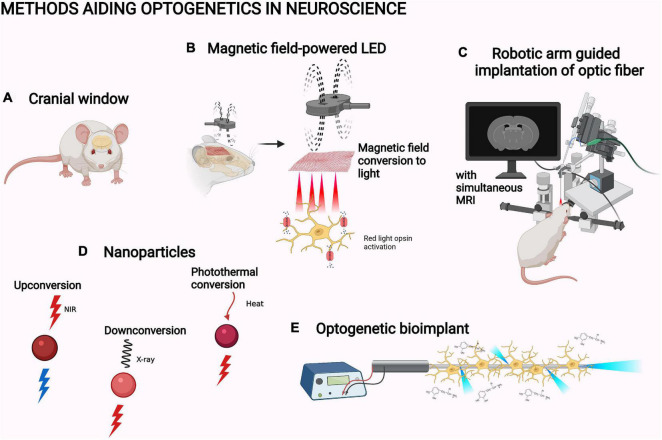
Methods aiding optogenetics in neuroscience. **(A)** Cranial window (Cha et al., 2020). **(B)** Magnetic field-powered LED (Lee et al., 2020). **(C)** Robotic arm guided implantation with magnetic resonance imaging, MRI (Chen et al., 2019). **(D)** Upconversion nanoparticle, UPNC (Wang et al., 2019), X-ray downconverting nanoparticle (Chen et al., 2021), and photothermal-converting nanoparticle (Chen X. et al., 2020; Qiao et al., 2021). **(E)** Optogenetic bioimplant (Vasudevan et al., 2019). The objects are not to scale. The figure was created with BioRender.com.

The illumination of optogenetic systems can be improved by advances in the light path or the light application. In neuroscience, cranial windows are the main way to clear the light path (Figure 5A). Cranial windows are based on replacing the skull with a transparent material or increasing the penetration of the skull by thinning it (Augustinaite and Kuhn, 2020). Both approaches suffer from the worsening of the optical quality of the window due to inflammation and regrowth (Yang et al., 2010; Kim et al., 2016). Glass windows also preclude further tissue access, for example, for a viral injection (Kim et al., 2016). An improvement of accessibility has been made through the development of glass windows with self-sealing silicone access ports (Roome and Kuhn, 2014). As another partial solution, the cranial window can nowadays be made from transparent polymers (Ruiz et al., 2013). They are flexible and chemically inert structures that are thus better suited for the purpose than glass. For example, a polydimethylsiloxane window for mice has been reported in *bioRxiv*: the window is removable, which is optimal for further experimentation with invasive implants (Figure 5A; Cha et al., 2020).

As an alternative for removing a piece of the skull and replacing it with a transparent material, the bone itself can be altered to increase light penetration. Optical clearing is a technique based mainly on the chemical alteration of the optical properties of the skull. The main objective is to minimize scattering of light by removing the highly refractive molecules and homogenizing of the tissue (Yu T. et al., 2021). The skull clearance protocol by Zhao et al. (2017) utilizes breakage of collagens, chelation of calcium, and replacement of water. The cleared skull enabled two-photon imaging of the microvasculature and the cortical dendrites in head-fixed mice. Interestingly, the clearing could be quickly reversed and then reapplied (Zhao et al., 2017). Iijima et al. (2021) have fascinatingly applied optical clearing of the brain *in vivo* without surgery. In their approach, called MAGICAL, the oral application of glycerol enabled improved imaging of hippocampal structures (Iijima et al., 2021).

In neuroscience, most studies have applied light through intracranial optical fibers. Use of robotic manipulator arms guided with simultaneous MRI has increased the accuracy of their implantation into rodent brain (Figure 5C; Chen et al., 2019). Instead of relaying the light via optic fibers, LED lights directly implantable in the experimental animal have also been developed. For example, Lee et al. (2018) slid a flexible LED based on semiconductor membrane technology under the skull in mice to directly illuminate the cortex (Figure 5B; Lee et al., 2018). NIR light illumination by this LED successfully activated the red-light opsin Chrimson in the mouse motor cortex. This induced movement in the whiskers and forelimb with related electromyography signals (Lee et al., 2018). Some exciting physical phenomena have also been utilized in developing specific LED designs for *in vivo* optogenetics: MMTENG is the first optogenetically utilized LED able to turn the energy of magnetic fields into electricity. The system can harvest energy from the stray magnetic fields created by surrounding electrical devices. This alleviates the limitation of battery life in long-term optogenetic studies. MMTENG has been utilized for inducing whisker movement with Chrimson (Lee et al., 2020). In a similar manner, a photovoltaic LED is able to turn light energy into electricity. Jeong et al. (2021) utilized an illumination system based on a subcutaneous NIR light sensor which transforms the energy of the light into an electric current used to power an intracranially implanted LED. This enables completely wireless, constant optogenetic control of whisker movement in behaving mice (Jeong et al., 2021).

Intracranially implanted lenses can be used to illuminate and image deep brain structures. Gradient index (GRIN) lenses are routinely utilized for this purpose in neuroscience (Jennings et al., 2019). In practice, the GRIN lenses are small glass cylinders that can be mounted on the brain surface or pushed into the brain tissue after making a tract for the lens. GRIN lenses have a gradual variation in their refractive index (i.e., light travels at different speeds in different parts of the lens). This enables modification of the lenses into unusual shapes and refraction of light (Gordon et al., 2011; Miyamoto and Murayama, 2016). GRIN lenses have been used to activate the red opsin ReaChR in neurons of the mouse visual cortex. The activation has been imaged in 3D as the system allowed digital holographic display of the neurons (Accanto et al., 2019).

The simultaneous use of other neuroscientific research applications can also disrupt illumination. The electrical activity of the brain is one of the fundamental parameters monitored in neuroscience. Combining optogenetics and electrophysiology enables simultaneous manipulation and recording of neural activity. Intracranial EEG provides better sensitivity and precision compared to the traditional electrophysiology methods (Parvizi and Kastner, 2018). For this, the EEG probe as well as the light source have traditionally needed to be fitted inside the brain tissue. Optrodes have been developed which combine both in one implant (Dufour and De Koninck, 2015; Patel et al., 2020). However, in addition to pure practical limitations in terms of space and tissue damage, there are problems that arise from the interaction of the electrical activity and light. Namely, shining light in the brain causes a photo-electric artifact in electrophysiological recordings when a traditional metal electrode is used (Dufour and De Koninck, 2015). This artifact can be avoided by using glass electrodes (Ono et al., 2018). Still, considerable technical developments are needed to enable cost-effective, efficient, and artifact-free recording deep in the brain with simultaneous optogenetic stimulation. The better penetration of red light optogenetics could possibly be used to ease these approaches in the future.

For topical parts of the brain, transparent intracranial EEG probes have been developed to be compatible with optogenetics (Yang et al., 2020; Tian J. et al., 2021). For example, an acrylic template with gold electrodes has been utilized with opsin-based neural mapping in mice (Seo et al., 2020). The flexibility of transparent intracranial EEG probes has also been improved. This enables more precise measurements as the effective contact area on the brain surface increases (Yang et al., 2020; Tian J. et al., 2021). These topical probes could be possibly morphed with intracranial LEDs to create similar solutions as optrodes.

The first microwell high-throughput method for optogenetics has the potential to streamline future research. The LED-platform, optoPlate-96, enables a simultaneous and independent control of 96 3-color optogenetic experiments in 96- or 384-well plates. The system has been used in a two-system study of proliferation and survival-signaling pathways with the plant phytochrome-based OptoSOS system (Bugaj and Lim, 2019); optoPlate-96 is an affordable tool, and the later addition of the graphical user interface will facilitate its future use (Thomas et al., 2020).

Other devices and programs can have a similar future in optogenetic neuroscience. For example, an extremely affordable LED controller, LED Zappelin’, eases optogenetic experiments during simultaneous two-photon imaging. The system has been utilized for optogenetic circuit mapping. *Drosophila melanogaster* larvae olfactory neuron activation by Chrimson has been visualized in the neurons by two-photon calcium imaging (Zimmermann et al., 2020). Specific devices are not a necessity, however. For example, optimization of the illumination to suit the specific optogenetic tool can be as effective; optimization of illumination has been done to match the kinetics of the red light-sensing anion opsin eNpHR3.0 (Zhang et al., 2019). A smartphone can work as well, as shown by Meloni et al. (2020). *Drosophila melanogaster* larvae expressing Chrimson in their nociceptive neurons were placed on top of the smartphone screen on an agarose sheet. Different maze-like light patterns were created on the screen by a specific app. The larvae avoided the light parts of the screen in the specific patterns. Therefore, animal movement has been controlled by a smartphone (Meloni et al., 2020).

Upconversion nanoparticles (UPNC) have been one of the major improvements for optogenetic illumination (Figure 5D; Wang et al., 2019). UPNC are capable of anti-Stokes process, that is, the ability to absorb at least two photons of lower energy to emit light at higher-energy wavelengths (Auzel, 1966). The ability to convert NIR light into blue light enables the use of blue light-activated optogenetic systems with the higher penetration of NIR light. Therefore, UPNC can help to circumvent the penetration problem. Upconversion systems can be based on upconverting rare earth metals or on triplet–triplet annihilation upconversion from organic chromophores (Zhu et al., 2019; Fallon et al., 2020). Both systems are constantly being further developed. For example, new inorganic UPNC have been characterized and the conditions for their optimal use have been studied (Krajnik et al., 2020; Skripka et al., 2020). Organic UPNC have been formed into hydrogels, which has been utilized in a 3D cell model and *in vitro* hippocampal neurons to activate blue light optogenetic systems (Sasaki et al., 2019; Meir et al., 2021).

Upconversion nanoparticles have other interesting characteristics which can be helpful in future research. For example, they have been successfully utilized as transfection vectors for an optogenetic apoptosis system without any transfection agents. In the same study, mouse tumors treated by the system have been monitored by an MRI based on the nanoparticles (Zheng et al., 2017). UPNC have been utilized for mice locomotion control with opsins through transcranial illumination. In the study, UPNC maintained their function in the limbic dorsal striatum for 2 months, which indicates the possibility for long-term optogenetic studies (Miyazaki et al., 2019). The utilization of different opsins with UPNC in several other neuroscientific targets has also been demonstrated (Chen et al., 2018). The careful design of UPNC allows the parallel use of optogenetic proteins activated with different wavelengths with a single illumination pulse. For example, this approach has been applied to an experiment where the same NIR pulse has activated both the red opsin Chrimson and the blue photosensitizer miniSOG. This system, based on the simultaneous increase in oxidative stress and cellular calcium, has been used to resolve the function of the motor neuron of *Caenorhabditis elegans* (Zhang Y. et al., 2020).

The penetration of light into biological tissues is still relatively limited for larger animal models, like non-human primates. The optogenetic manipulation of structures deep in the primate brain is not possible without an intracranial probe, not even with NIR light (Jacques, 2013). X-rays can pass through biological tissues, but ionizing radiation can be harmful for the subject (Wang et al., 2009). Still, X-ray imaging is a common practice in medicine. Therefore, downconverting nanoparticles could be the next solution for the penetration problem. One such system can convert X-rays into red light (Figure 5D; Chen et al., 2021). This system has been used to activate motor neurons in the primary motor cortex of mice with the red light-sensing opsin ReaChR (Chen et al., 2021).

### Other Methods

Besides the advances in transfection and illumination, various other methods have facilitated the use of red light in optogenetics. Data-driven approaches and simulations are just one example: red-shifted opsins have been found through machine learning-based data mining, and the photocycle of Chrimson has been theoretically optimized through a mathematical model (Gupta et al., 2019; Inoue et al., 2021). Similarly, local illumination enhancement through aluminum nanostructures has been simulated for the possible future use of lower overall illumination intensities (Keck et al., 2021).

Heat-responsive promoters could be another tool to be utilized. They are parts of DNA that can bind heat-activated transcription activators, then activate the gene transcription. They have been utilized with the NIR light absorbance of photothermal-converting nanoparticles, which convert light to heat. In novel systems, an HSP70 promoter has been utilized *in vivo* to decrease the tumor size of mice (Figure 5D; Chen X. et al., 2020; Qiao et al., 2021). The earlier system has been a novel photothermal CRISPR system, nanoCRISPR (Chen X. et al., 2020). Its simultaneous utilization with optogenetic CRISPR systems could offer interesting applications in neuroscience. The other has been a pro-apoptotic, expression-based apoptosis system (Qiao et al., 2021). Similarly, it could be utilized alongside UPNC-related apoptosis systems to ensure thorough ablations.

Different platforms have also created new opportunities for red light-based optogenetics. Microfluidic biochips have been used to study axon development. There, a small 3D gel environment on the chip has enabled the co-culturing of motor neurons with main peripheral glial cells, Schwann cells. The opsin activation of the motor neurons has increased the axon growth and myelination by the Schwann cells (Hyung et al., 2019).

An optogenetic bioimplant has also been created (Figure 5E; Vasudevan et al., 2019). The implant consists of a leaky optic fiber and a chronoamperometer made from pyrolytic carbon. Pyrolytic carbon enables the growth and the differentiation of the opsin-expressing neural stem cells into dopaminergic neurons. The neurons are evenly activated by illumination through micro-fragments on the fiber surface. The activation can be measured due to changes in the electrical properties of the implant, which are based on the oxidation of the released dopamine on the fiber surface. The implant has not been utilized *in vivo* but could be an interesting tool for the research of Parkinson’s disease (Vasudevan et al., 2019).

Organoids are organ-mimicking multicellular 3D structures. Retinal organoids have been utilized for optogenetic vision restoration in mice by Garita-Hernandez et al. (2019, 2021). In their studies, human-induced pluripotent stem cells were differentiated into retinal organoids. The cone cells, expressing red light opsin Jaws, were separated from the organoids and delivered into the subretinal space of mice eyes *in vivo*. Consequently, the cone cells formed synapses with the natural preceding signaling cells of the visual pathway. Per the natural ON/OFF pathway of the signal transmission of sight, natural cone cells constantly inhibit their preceding signaling cells in the dark, and light inhibits this constant inactivation. Here, the natural signaling cells were activated after the inactivation of the modified cone cells by red light-induced Jaws activity (Garita-Hernandez et al., 2019, 2021). This application indicates that various *in vivo* functions may be restored through the transplantation of red light optogenetic cells.

## Discussion

In our review, we have focused on the current state and advancement in red light optogenetics in neuroscience. We have outlined the physical and biological properties to be considered regarding illumination, and we address the advantages of red light for the light penetration problem. We have also reviewed the current state of the research on red light-sensing optogenetic actuators. Several interesting optogenetic systems have recently been introduced, and we are keen to see their future applications. Lastly, we have highlighted different methods that support the future of optogenetic research. The methods have been shown to originate from a multitude of closely related fields of science with the primary focus on the improvements in transfection and illumination methods. Overall, we hope that our review will facilitate the use of red light for optogenetics in neuroscience.

We would finally like to leave the reader with a list of hints and suggestions for future studies, which is based on the reviewed literature:

• Utilize the better tissue penetration of red light in transcranial illumination.
• Apply the photochromaticity of phytochromes and CBCRs for reversible biological events.
• Improve existing heterodimerization systems with the recent smaller binding partners.
• Apply the recently found opsin types for novel optogenetic applications.
• Utilize the two-photon absorption of bacterial phytochromes for better tissue penetration.
• Create new multichromatic systems with a wider range of actuators and wavelength ranges.
• Transfer the red light-sensing optogenetic systems published *in vitro* to neuroscientific applications *in vivo*.
• Consider nanoparticles as a research tool and collaborate with someone with knowledge of their production and use.
• Use high-throughput *in vitro* platforms to streamline optogenetic studies.
• Consider the possibilities of optogenetic neural bioimplants for *in vivo* studies.
• Utilize the heat produced by the red light in heat-responsive approaches.
• Favor the use of one-vector approaches with smaller proteins, homodimerization systems, and linked opsins.
• Consider methods that help injection or implantation in the brain, like robotics or MRI.
• Prefer the use of transcranial and topical intracranial approaches in recording and imaging of the optogenetic effect, which preserves the brain under study.
• Turn computer-based results about red light-driven systems into actual applications.
• Note the possible effects of endogenous opsins and PBM on the optogenetic experiments.
• When plant phytochromes and CBCRs are used in the brain, ensure their chromophore availability.
• Facilitate the simultaneous use of EEG and optogenetic applications in the deep brain by morphing intracranial LEDs with intracranial transparent EEG probes.
• Expand the minimalistic optogenetic toolbox of CBCRs to benefit from their small size and photochromatic activity.

## Author Contributions

KL, HT, and MN designed and wrote the manuscript. All the authors contributed to the article and approved the submitted version.

## Conflict of Interest

The authors declare that the research was conducted in the absence of any commercial or financial relationships that could be construed as a potential conflict of interest.

## Publisher’s Note

All claims expressed in this article are solely those of the authors and do not necessarily represent those of their affiliated organizations, or those of the publisher, the editors and the reviewers. Any product that may be evaluated in this article, or claim that may be made by its manufacturer, is not guaranteed or endorsed by the publisher.

## Abbreviations

AAV: adeno-associated virus
CBCRs: cyanobacteriochromes
GRIN: gradient index
EEG: electroencephalography
PBM: photobiomodulation
PDB: Protein Data Bank
PIF: phytochrome-interacting factor
NIR: near-infrared
MIR: magnetic resonance imaging
UPNC: upconversion nanoparticles.
